# A severe 2017 influenza season dominated by influenza A(H3N2), Victoria, Australia

**DOI:** 10.5365/wpsar.2018.9.5.010

**Published:** 2018-09-28

**Authors:** KA Grant, KS Carville, SG Sullivan, J Strachan, J Druce, JE Fielding

**Affiliations:** a Victorian Infectious Diseases Reference Laboratory, Melbourne, Australia.; b WHO Collaborating Centre for Reference and Research on Influenza, Melbourne, Australia.; c School of Global and Population Health, University of Melbourne, Australia.; d Discipline of General Practice, University of Adelaide, Australia.; e Communicable Diseases Epidemiology and Surveillance, Health Protection Branch, Department of Health and Human Services.; f School of Global and Population Health, University of Melbourne, Australia.

## Abstract

Surveillance for influenza-like illness (ILI) and laboratory-confirmed influenza in Victoria, Australia is undertaken jointly by the Victorian Infectious Diseases Reference Laboratory and the Victorian Government Department of Health and Human Services from May to October each year. Surveillance data comprise notifiable laboratory-confirmed influenza and ILI reporting from from two sources – a general practice sentinel surveillance programme and a locum service.

The magnitude of the 2017 influenza season was high in Victoria with widespread circulation of influenza type A(H3N2), which peaked in September. A record number of laboratory-confirmed influenza cases were notified, and the proportion of ILI cases to total consultations from both the general practice and locum service were higher than previous years. Notified cases of influenza A were older than influenza B cases with 25% compared to 17% aged more than 65 years, respectively. The proportion of swabs that were positive for influenza peaked at 58%. Antigenic characterization suggested a good match between the circulating and vaccine strains of influenza A(H3N2).

Most of the increases observed in notified cases of laboratory-confirmed influenza in recent years in Victoria have been attributed to increases in testing. However, that cases of ILI also increased in Victoria in 2017 is suggestive that 2017 was a relatively severe season. The dominance of influenza type A(H3N2), the extended duration of elevated activity, and a potential phylogenetic mismatch of vaccine to circulating strains are likely to have contributed to the relative severity of the 2017 season.

Victoria is Australia’s second most populous state and is the mainland’s southernmost state. It has a temperate climate with an influenza season usually occurring in the cooler months between May and October. The Victorian Infectious Diseases Reference Laboratory (VIDRL), in partnership with the Victorian Government Department of Health and Human Services (DHHS), coordinates influenza-like illness (ILI) and laboratory-confirmed influenza surveillance in Victoria. There are three data sources included in the influenza surveillance system.

The Victorian Sentinel Practice Influenza Network (VicSPIN) is a surveillance programme of sentinel general practitioners (GPs) that monitors ILI and laboratory-confirmed influenza in the community (previously known as the Victorian General Practice Sentinel Surveillance system). (*1*) VicSPIN operates annually between May and October. (*1*) Samples collected from ILI patients that subsequently test positive for influenza by VIDRL are submitted to the World Health Organization (WHO) Collaborating Centre for Reference and Research on Influenza for strain characterization and antiviral drug sensitivity testing.

Notified laboratory-confirmed influenza cases are reported from medical practitioners and laboratory services in Victoria who are required by law to notify DHHS of all laboratory-confirmed cases of influenza within five days of diagnosis. Notifications require identification, demographic and diagnostic data.

The National Home Doctor Service (NHDS) is the largest medical locum service in Australia and provides 24-hour medical services to patients at their residences. (*2*) The data entered into the NHDS database were analysed to determine the proportion of ILI diagnoses made from all consultations.

In this study, the data from these three surveillance programmes are used to describe the epidemiology of the 2017 influenza season in Victoria, Australia.

## Methods

### Surveillance data

#### Victorian Sentinel Practice Influenza Network (VicSPIN)

In 2017, 88 GPs participated in the VicSPIN surveillance programme from 1 May to 4 November. GPs reported the number of ILI cases each week and the total number of consultations as well as age, gender and vaccination status of ILI cases. The definition of ILI was a patient with fever, cough and fatigue/malaise. A nose or throat swab was collected from as many ILI cases as possible, at the GPs discretion, for those patients presenting within four days of symptom onset. Additional data collected on swabbed patients included seasonal influenza vaccination status for the previous year (as well as the current year), date of vaccination/s, fever (reported or measured) and any co-morbidity for which influenza vaccination is recommended. (*3*)

The samples were submitted to VIDRL where ribonucleic acid was extracted and tested using in-house validated real-time multiplex polymerase chain reaction (PCR) assays to detect type A influenza viruses (matrix gene), type B influenza viruses (nucleoprotein gene) and type C influenza viruses (matrix gene). Influenza A virus-positive samples were further subtyped using individual real-time PCR assays incorporating primers and probes specific for the haemagglutinin gene of A(H1N1)pdm09 and A(H3) strains. (*4*) Samples positive for influenza were forwarded to the WHO Collaborating Centre for Reference and Research on Influenza for antigenic characterization.

#### Notifiable diseases surveillance

Cases of laboratory-confirmed influenza notified to DHHS in 2017 were extracted from the DHHS system. Only cases routinely notified were included in the analysis; cases identified and reported as part of an outbreak investigation or from other screening activities were excluded.

#### National Home Doctor Service (NHDS)

NHDS locums entered consultation data into the NHDS database daily. De-identified data from this database were accessed by VicSPIN staff who applied an algorithm to return records in which the words “influenza” and “flu” were included in the case notes. These records became the ILI cases and, along with total consultations, were aggregated daily and made available to the researchers via a secure web site. To avoid inclusion of those immunized prophylactically during the 2009 pandemic, records that contained the terms “Fluvax,” “at risk” and “immunization” were excluded.

### Strain characterization and antiviral resistance testing

All influenza-positive samples in Victoria, including those from VicSPIN, were sent to the WHO Collaborating Centre for Reference and Research on Influenza for antigenic characterization and antiviral drug sensitivity testing. Samples were first inoculated into Madin-Darby Canine Kidney cells to obtain virus isolates. Those successfully isolated were then analysed by haemagglutination inhibition assay to determine antigenic similarity to the current vaccine strains. (*5*, *6*) Isolates were identified as antigenically similar to the reference strain if the test samples had a titre less than an eightfold difference compared with the homologous reference strain. Isolates were also tested in a neuraminidase inhibition assay to determine susceptibility to the antiviral drugs oseltamivir, zanamivir, peramivir and laninamivir.

### Data analyses

Descriptive analyses of the surveillance data were conducted in Microsoft Excel. Comparison of proportions were tested using the χ2 test in Stata (version 14.1; StataCorp LP, College Station, TX, USA) with *P* < 0.05 considered significant.

The WHO method for ILI thresholds (*7*) was used to assign three threshold levels: seasonal (4–15 ILI cases per 1000 consultations), average (15–24 ILI cases per 1000 consultations) and alert thresholds (> 24 ILI cases per 1000 consultations). Data from previous years were compared to evaluate the magnitude of the 2017 season.

## Results

### Influenza-like illness

During 2017, VicSPIN GPs conducted 151 618 consultations of which 1208 were for patients with ILI – a proportion of 8.0 ILI cases per 1000 consultations. The NHDS reported 206 833 consultations of which 4512 were for ILI, giving a proportion of 21.8 ILI cases per 1000 consultations.

**Figure 1:**
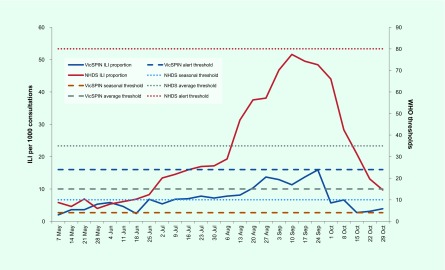
**VicSPIN and NHDS ILI proportion and WHO thresholds, Victoria, Australia, 2017**

The proportion of ILI cases reported by VicSPIN was within the average activity thresholds from 25 June to 8 October and peaked in late September at 15.9 cases per 1000 consultations to the alert threshold (**Fig. 1**). The majority of ILI cases were aged in the working age groups, mostly in the 30–49 age group (29.6%). Only 5.5% were aged 0–4 years and 11.1% were aged over 65 years.

[**insert** Figure 1]

**Figure 2:**
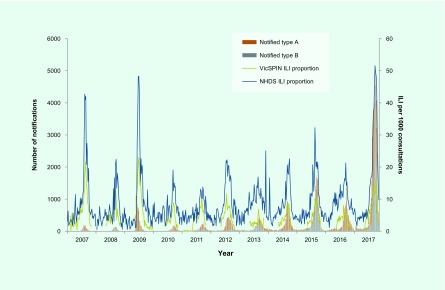
**VicSPIN and NHDS ILI proportions, Victoria, Australia, 2007 to 2017**

The proportion of ILI cases reported by NHDS peaked in early September at 51.6 per 1000 consultations (**Fig. 1**). ILI activity was within the above-average activity threshold from mid-August to the end of September; it was above the lower limit for average activity on either side of this peak from mid-July to mid-October. The peaks for both VicSPIN and NHDS were higher than all previous years (**Fig. 2**).

[**insert** Figure 2]

### Notified laboratory-confirmed influenza

**Figure 3:**
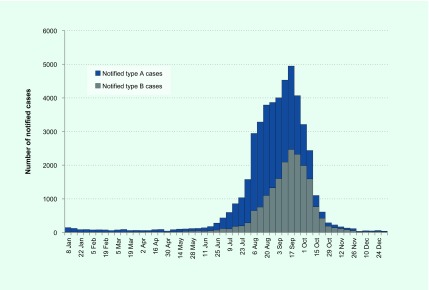
**Notified cases by influenza type, Victoria, Australia, 2017**

**Figure 4:**
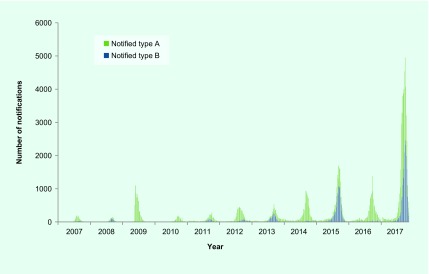
**Notified cases of laboratory-confirmed influenza by influenza type, Victoria, Australia, 2007 to 2017**

There were 47 133 cases of laboratory-confirmed influenza routinely notified to DHHS in 2017 (**Fig. 3**). Of the 2017 cases, 64% were type A and 35% were type B. Ninety-five per cent (*n* = 44 796) of cases were notified during the usual influenza season of 1 May to 4 November. Notifications of influenza A peaked in August, whereas notifications of influenza B peaked later in September (**Fig. 3**). The number of notifications for 2017 was higher than previous years (**Fig. 4**).

[**insert** Figure 3]

[**insert** Figure 4]

The modal age group of notified influenza A cases was 65 years (*n* = 6866; 25%); for influenza B it was 30–49 years (*n* = 4864; 25%) (**Table 1**).

**Table 1:** Notified and VicSPIN-detected laboratory-confirmed influenza cases, by age group and type/subtype, Victoria, Australia, 2017

-	-	A(H1)	A(H3)	A (not subtyped)	B
-	**Age group (years)**	***n*(%)**	***n*(%)**	***n*(%)**	***n*(%)**
**Notified cases**	**0–4**	**-**	**-**	**2565 (9%)**	**1352 (7%)**
-	**5–14**	**-**	**-**	**3247 (12%)**	**4044 (21%)**
-	**15–29**	**-**	**-**	**3945 (14%)**	**2579 (14%)**
-	**30–49**	**-**	**-**	**6604 (24%)**	**4864 (25%)**
-	**50–64**	**-**	**-**	**4555 (16%)**	**3083 (16%)**
-	**^3^ 65**	**-**	**-**	**6866 (25%)**	**3180 (17%)**
**VicSPIN**	**0–4**	**3 (8%)**	**2 (1%)**	**-**	**2 (2%)**
-	**5–14**	**8 (22%)**	**12 (8%)**	**-**	**17 (17%)**
-	**15–29**	**6 (17%)**	**33 (23%)**	**-**	**17 (17%)**
-	**30–49**	**8 (22%)**	**48 (33%)**	**1 (50%)**	**42 (41%)**
-	**50–64**	**8 (22%)**	**33 (23%)**	**1 (50%)**	**18 (17%)**
-	**^3^ 65**	**3 (8%)**	**18 (12%)**	**-**	**7 (7%)**

### VicSPIN laboratory-confirmed ILI cases

Sixty per cent (*n* = 725) of the 1208 cases of ILI reported through VicSPIN were swabbed. Of these, 40% were positive for influenza: 12% were influenza A(H1N1)pdm09, 51% were influenza A(H3N2), and 37% were influenza B.

**Figure 5:**
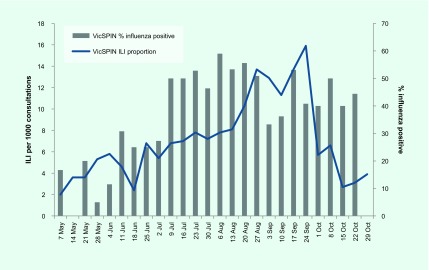
**VicSPIN influenza-positive cases, Victoria, Australia, 2017**

The majority of laboratory-confirmed influenza cases reported through VicSPIN (75%) were of working age (15–65 years) (**Table 1**). Eighteen of the 28 cases (64%) reported in those aged 65 years or older were type A(H3N2). Most influenza cases were detected between 10 July and 24 September (*n* = 232; 81%). The percentage of VicSPIN swabs positive for influenza peaked in July and was elevated until early October (**Fig. 5**) when cases of influenza type A decreased.

[**insert** Figure 5]

**Figure 6:**
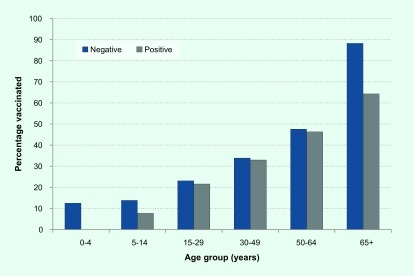
**Percentage of ILI cases vaccinated by influenza status and age group, VicSPIN, 2017**

Vaccination status was reported for 91% of the 725 swabbed patients; of these, 35% were vaccinated with the proportion vaccinated increasing with age (**Fig. 6**). The difference in the proportion of influenza-positive and influenza-negative ILI cases who were vaccinated was statistically significantly (32% and 40%, respectively; *P* = 0.02). However, when the data were stratified by age, the difference was only statistically significant for those aged 65 years and older (64% and 88%, respectively; *P* = 0.01).

[**insert** Figure 6]

Of the 725 swabs received through VicSPIN, 18.2% were from patients reported to have co-morbidities for which influenza vaccine is recommended. Of these, 38.6% were positive for influenza, 22.7% were positive for other respiratory viruses and 38.6% were negative for any respiratory virus. Almost two thirds of these patients with co-morbidities (67%; *n* = 88) were vaccinated. The most commonly reported co-morbidity was asthma (*n* = 24; 18%).

### Strain characterization and antiviral resistance testing

There were 1675 influenza isolates characterized antigenically in 2017 in Victoria (**Table 2**). A neuraminidase inhibition assay was conducted on 2378 isolates, with two being resistant to oseltamivir, one influenza A(H1N1)pdm09 and one influenza A(H3). One influenza A(H1N1)pdm09 was also resistant to zanamivir.

**Table 2:** Victorian influenza isolates typed by haemagglutination inhibition assay at the WHO Collaborating Centre for Reference and Research on Influenza, VIDRL, 2017

Strain	*n*(%)
**A(H3)/Hong Kong Special Administrative Region/4801/2014**	**678 (41%)**
**B/Phuket/3073/2013**	**606 (36%)**
**A/Michigan/45/2015 (H1N1)pdm09**	**347 (21%)**
**B/Brisbane/60/2008**	**44 (9%)**

## Discussion

Victoria experienced a relatively severe influenza season in 2017; the seasonal peaks for both the ILI and laboratory-confirmed components of the system were the highest since the pandemic year of 2009. The ILI proportions from both VicSPIN and NHDS showed above-average activity thresholds. Since 2009, large annual increases in notified cases of laboratory-confirmed influenza have been largely attributed to increased laboratory testing, as ILI proportions reported from VicSPIN and NHDS remained comparable in magnitude. (*8*, *9*) However, the increase observed for notified cases in 2017 was particularly large at almost three times higher than the next largest year in 2015, and seven times more than the pandemic year of 2009, and was coupled with increases in ILI proportions reported from VicSPIN and NHDS. Similarly, the proportion of swabs positive for influenza in VicSPIN during 2017 was 41%, higher than previous seasons where it ranged from 22% to 39% (median = 34%) from 2010 and 2016. (*8*) A higher number of cases than usual reported to DHHS during summer 2017 also contributed to the overall increase in notifications. The high magnitude of the 2017 influenza season was also observed in other states in Australia (*10*) with a similar increase reported in laboratory-confirmed influenza notifications nationally. (*11*)

The 2017 influenza season in Victoria was dominated by circulation of influenza A(H3N2) with an increase in influenza B later in the season. This was similar in New Zealand (*12*) and Western Australia (*13*) for their 2017 season and the United States of America (*14*) and Canada (*15*) for their 2017–18 seasons. The relative severity of the Victoria season could be explained by the dominance of influenza A(H3N2). This subtype disproportionately affects older age groups, while influenza B is more common in younger age groups. (*16*) While a large proportion of notified cases of influenza A were not subtyped, those aged ≥ 65 years comprised the highest proportion of influenza A notified cases, and the median age of influenza A cases was higher than for influenza B cases. The percentage of VicSPIN influenza cases typed as A(H3N2) was highest in the ≥ 65 years age group compared to A(H1).

The strains included in the 2017 quadrivalent influenza vaccine were A/Michigan/45/2015 (H1N1)pdm09-like virus; A/Hong Kong Special Administrative Region/4801/2014 (H3N2)-like virus; B/Brisbane/60/2008-like virus; and B/Phuket/3073/2013-like virus. (*17*) The antigenic characterization datum from the Victorian 2017 season suggested a good match between the influenza A(H3N2) vaccine and these circulating strains; however, interim analysis of Australian data (including VicSPIN data) showed a low effectiveness of the 2017 influenza vaccine against influenza A(H3N2) infection of 10% [95% confidence interval (CI): –16 to 31]. (*18*) This may partially explain the higher number of influenza notifications in Victoria in 2017, but also serves to highlight the limited value of antigenic characterization. Phylogenetic typing of virus isolates may be more useful to assess the degree of match between circulating and vaccine strains.

Low vaccine effectiveness against influenza A(H3N2) is a persisting problem, speculated to be caused by genetic changes in vaccine virus haemagglutinin arising during passage in eggs, resulting in egg-derived viruses that are different from the cell reference strains. In response and to improve vaccine effectiveness in the elderly in 2018, two higher-immunogenicity trivalent influenza vaccine formulations (one a high-dose vaccine and another containing an adjuvant) will be funded in Australia under the National Immunization Program for those aged ≥ 65 years. (*19*)

Cases presenting with co-morbidities to GPs had a lower proportion positive for influenza than those without co-morbidities. This may be due to the higher influenza vaccination rates in this group at 66.7% as compared to 35.4%. GPs were also encouraged to test as many patients as possible in 2017 through the VicSPIN programme, so those with co-morbidities, such as asthma, may have been swabbed more than in previous seasons.

The influenza surveillance system in Victoria has several limitations including the lack of subtyping in the notifications data, variable age-structures between data sources and variable sensitivity of VicSPIN and NHDS ILI case detection. The NHDS is more sensitive due to the different search algorithms. Most ILI cases that presented to GPs were of working age, especially the 15–29 and 30–49 years old, which may relate to requirements for sick certificates for workplaces and universities. However, the lack of subtyping information for the notifications data limits the ability to determine if subtypes seen in VicSPIN are representative of those seen in the different age groups that are more likely to be notified than those detected in GP sentinel surveillance. While hospital-based surveillance of influenza has not been included in this report, these data are also used by DHHS to further understand influenza epidemiology throughout the season. (*20*)

The varied data sources used for influenza surveillance in Victoria provide a comprehensive overview of influenza and ILI. The comparison of ILI activity and notifications over time allows a more nuanced understanding of the season than analysing notifications alone and provides the evidence to suggest that the 2017 influenza season in Victoria was more severe compared with previous seasons.
